# Genome Sequences of Industrially Relevant *Saccharomyces cerevisiae* Strain M3707, Isolated from a Sample of Distillers Yeast and Four Haploid Derivatives

**DOI:** 10.1128/genomeA.00323-13

**Published:** 2013-06-06

**Authors:** Steven D. Brown, Dawn M. Klingeman, Courtney M. Johnson, Alicia Clum, Andrea Aerts, Asaf Salamov, Aditi Sharma, Matthew Zane, Kerrie Barry, Igor V. Grigoriev, Brian H. Davison, Lee R. Lynd, Paul Gilna, Heidi Hau, David A. Hogsett, Allan C. Froehlich

**Affiliations:** Biosciences Division, Oak Ridge National Laboratory, Oak Ridge, Tennessee, USAa; BioEnergy Science Center, Oak Ridge, Tennessee, USAb; US Department of Energy Joint Genome Institute, Walnut Creek, California, USAc; Mascoma Corporation, Lebanon, New Hampshire, USAd; Thayer School of Engineering, Dartmouth College, Hanover, New Hampshire, USAe

## Abstract

*Saccharomyces cerevisiae* strain M3707 was isolated from a sample of commercial distillers yeast, and its genome sequence together with the genome sequences for the four derived haploid strains M3836, M3837, M3838, and M3839 has been determined. Yeasts have potential for consolidated bioprocessing (CBP) for biofuel production, and access to these genome sequences will facilitate their development.

## GENOME ANNOUNCEMENT

*Saccharomyces cerevisiae* is the first eukaryote for which a genome was completely sequenced (1). It has been studied intensely for decades as a model organism, is generally recognized as safe (GRAS), is used as a host for recombinant protein production (2), and is tolerant of a wide range of physiological stresses, such as low pH, high ethanol, and high osmotic stress (3, 4, 5). *S. cerevisiae* is used in the baking industry and fermentations for food-grade alcohol production, its pentose utilization capabilities have been developed (3, 6), and yeasts have potential for consolidated bioprocessing (CBP) for biofuel production (7, 8). The 12-Mb *S. cerevisiae* S288c genome encodes approximately 6,000 open reading frames, and the organism can maintain stable haploid or diploid states (9). To better understand *S. cerevisiae* and its potential for CBP development, we generated the genome sequences for the diploid strain M3707 isolated from a sample of distillers yeast and four haploid derivatives named M3836, M3837, M3838, and M3839.

The genomes of *S. cerevisiae* strain M3707 and strains M3836, M3837, M3838, and M3839 were sequenced using the Illumina (10) HiSeq DNA sequencing platform according to the manufacturer’s instructions. Long mate-pair and paired-end (PE) libraries were used to sequence genomic DNA for each strain, with insert sizes of ~4 kb and ~270 bp, respectively. In addition, cDNA derived from RNA that represented 17 different growth conditions was sequenced from a ~270-bp insert size PE library to aid gene model predictions. Filtered reads were assembled with AllPaths-LG (release version R41554) (11). Each strain has an estimated genome size of approximately 11.5 Mb, with a mean genome sequence coverage of ~149× across all strains. The genome sequences assembled into between 84 and 120 contigs and 43 scaffolds for each strain. Approximately 6,000 genes were predicted in each of the five strains using the Joint Genome Institute (JGI) annotation pipeline (12) that contained a set of gene predictors (13, 14, 15).

The genome sequences of the new strains and strain 288c are very similar, with 99.05 to 99.1% similarity between the genomes as measured by average nucleotide identity (ANI) (16). Near-perfect orthologs (≥98% coverage and ≥98% percent identity) are found for 90.4% of M3707 and 288c genes, and 97.4% of these are syntenic. Interestingly, over half of the predicted gene models are identical copies; the rest are affected by insertions and deletions. In comparison to 288c, M3707 lacks around 3.5% of sequence (~325 kb) containing 46 genes, while it carries an additional 1.7% (~150 kb) of sequence containing 33 genes. Most of the unique genes encode proteins of unknown function. However, an interesting exception is a gene containing a melibiose domain, which is conserved among the five strains that were sequenced as part of this study. *S. cerevisiae* strain M3707 and strains M3836, M3837, M3838, and M3839 are industrially relevant, and their genome sequences will provide a useful foundation for future metabolic engineering, systems biology, and comparative genomics studies to develop CBP *S. cerevisiae* strains.

### Nucleotide sequence accession numbers.

The genome sequences and annotations are available via the JGI fungal genomics resource MycCosm (http://jgi.doe.gov/fungi [17]) and have been deposited in DDBJ/EMBL/GenBank under the accession no. AMQB00000000, AMQC00000000, AMQD00000000, AMQE00000000, and AMQF00000000 for strains M3707, M3836, M3837, M3838, and M3839, respectively.
